# Micromassage Compression Leggings Associated with Physical Exercise: Pilot Study and Example of Evaluation of the Clinical and Instrumental Effectiveness of Conservative Treatment in Lipedema

**DOI:** 10.3390/life14070854

**Published:** 2024-07-08

**Authors:** Lorenzo Ricolfi, Valeria Reverdito, Guido Gabriele, Micaela Bortolon, Ilaria Macherelli, Piero Haag, Nicoletta De Santis, Massimo Guerriero, Laura Patton

**Affiliations:** 1 Endocrinology and Lymphology Clinic, 38096 Vallelaghi, TN, Italy; lorenzoricolfi@hotmail.it; 2 Human Project Studio, 10138 Torino, TO, Italy; valeria.reverdito@gmail.com; 3 Department of Medical Biotechnology, University of Siena, 53100 Siena, SI, Italy; guido.gabriele@unisi.it; 4 Rehabilitation Unit and Lymphology Clinic, Institute San Gregorio, 31049 Valdobbiadene, TV, Italy; micaelabortolon@gmail.com; 5 MH Fisio, 00159 Roma, RM, Italy; ilariamacherelli24@gmail.com (I.M.); pierohaag@gmail.com (P.H.); 6 Clinical Research Unit, IRCCS Sacro Cuore Don Calabria Hospital, 37024 Verona, VR, Italy; nicoletta.des93@gmail.com (N.D.S.); massimoal.guerriero@gmail.com (M.G.)

**Keywords:** lipedema, adipose tissue, pain, compression, micromassaging leggings, exercise, treatment, ultrasound

## Abstract

We evaluated the effect of compression leggings with micromassage in association with physical activity on women with lipedema, not previously treated and without recent changes in body weight. The treatment resulted in an improvement in all subjective parameters, in spontaneous and evoked pain, in the volume of the limbs, in the absence of significant changes in body weight, and regardless of the duration of use, age, years of illness, the clinical stage, and body mass index (BMI). Evoked pain did not improve in areas affected by untreated lipedema; foot circumferences did not increase. We found a significant reduction in the thickness of subcutaneous adipose tissue (SAT) and skin evaluated in multiple points of the lower limb. Micromassage compression leggings are proposed as an integral part of conservative treatment. A method is proposed for the clinical evaluation of evoked pain, called the Progressive Pain Check (PPC), which allows for the calculation of a numerical score called the Ricolfi–Patton Score (RPS) and for the ultrasound evaluation of tissues. The method is simple and repeatable and allows for completion of the clinical evaluation of the patient at diagnosis and for an evaluation of the effects of various treatments, even applied to just one side of the body.

## 1. Introduction

Lipedema is a chronic pathology that typically affects the female sex, often confused with lymphoedema, lipodystrophy, or obesity. The disease can potentially involve any part of the body, but it frequently affects the limbs, usually sparing the hands and feet: this often results in a typical disproportion between the extremities and the trunk, due to the localized and symmetrical increase in subcutaneous adipose tissue (SAT) in the affected areas [1,2,3]. The true prevalence of lipedema is not known with certainty, as large epidemiological studies are lacking, and the available studies report data that are sometimes unclear. This is because the studies relate to both female and male populations or are obtained from pre-selected populations, such as patients hospitalized or visited for suspected lymphological problems. Furthermore, there is a lack of homogeneity in the diagnostic criteria used in the studies. It should be noted that the estimated prevalence of lipedema as reported in international consensus documents varies considerably. For instance, the prevalence in children in the United States is estimated to be 6.5% (11% if only female children are considered), and in women in Germany, it is estimated to be between 6% and 8%. Furthermore, in vascular clinics, the prevalence is estimated to be between 15% and 19% [4]. Lipedema tissue differs from healthy adipose tissue by the presence of inflammation and fibrosis, and it is associated with spontaneous or provoked pain in which, in some cases, alterations in sensitivity are present in the affected area. The fibrotic component would seem to be responsible for the incongruous volumetric reduction in the affected limbs despite the weight loss obtained with nutritional measures, physical activity, or bariatric surgery [4].

Lipedema is identified by clinical exam with diagnostic criteria to help guide the clinical diagnosis, since there are no known biomarkers at the moment, nor are there any specific diagnostic tests [2,4]. Even if all consensus documents and international guidelines agree on the diagnosis based on clinical aspects, the clinical signs and symptoms indicated as useful for the purpose of diagnosis of lipedema are not always the same and are not measurable. There are five types of lipedema described, based on the affected body area. The anatomical locations of lipedema include the hips and buttocks (Type 1), the area from the umbilicus to the knees (Type 2), the area from the umbilicus to the ankles (Type 3), the arms (Type 4), and the lower legs (Type 5) [4]. The most frequent appears to be the Type 3 form [4].

Furthermore, based on appearance, there is another classification that differentiates lipedema into two forms: columnar and lobar. The first one is characterized by a typical cylindrical limb, whereas the second one consists of a limb showing large lobes of tissue protruding from the sides [5].

Although there is not yet complete agreement between scientific societies on the clinical criteria to be used, three stages can be used to describe lipedema’s clinical severity. One of the most commonly used stagings is the following [4]:

- Stage 1: loose fibrotic connective tissue organized in subdermal pebble-like structures can be felt by palpating a smooth-texture skin;
- Stage 2: more lipedema tissue and skin dimpling are detected, and more and larger palpable nodules may be found;
- Stage 3: increased lipedema tissue that appears more fibrotic in texture and with numerous large subdermal nodules and overhanging lobules of tissue.

When lymphological alterations arise, especially in advanced Stage 3, this condition is referred to as Stage 4 or lipo-lymphedema. 

If alterations in the lymphatic system arise and remain parallel to the lipedema (this condition can be detected in all stages, even in the early ones), some authors use the term lympho-lipedema [6].

### 1.1. Treatment

The treatment of lipedema is mainly divided into conservative treatment and surgical treatment. We will focus on conservative treatment, being the object of this work. The standard conservative therapy for lipedema includes compression therapy, specific physiotherapy and nutritional treatments, and specific physical activity plans. Objectives of conservative treatments are the elimination or management of symptoms, including management of pain, impaired mobility, edema, and psychosocial issues [2], and the prevention of complications (increase in volume; dermatological, lymphatic, and orthopedic complications) [2,3,4]. Compression therapy has always been and remains an important element of “best practice” in the treatment of patients suffering from lipedema [7], but the notion is increasingly emerging that it is fundamental to combine different treatments, such as body weight control, to reduce associated complications and obtain good results from other applied therapies [1,8]. All the main consensus documents and guidelines agree on the usefulness of compression therapy, but there is not complete agreement on which kind of compressive treatment to choose and how to apply it, nor on the indication, which can vary in general based on various factors. Compression therapy is recommended for the treatment of symptoms, for supporting distorted tissues and promoting mobility, for preventive purposes, and for reducing edema in lipo-lymphedema, and it can have an anti-inflammatory effect on the subcutaneous tissue [1,2,7]. Although not all international documents agree on the indication for the use of compression therapy with multilayer bandages, the use of elastic compression garments is generally recommended by all, with the exception of milder cases of lipedema, but also despite the fact that the studies that demonstrate its effectiveness on symptoms and clinics are currently very few. There is a difference in the indications regarding the methods and times of use of the stocking and the choice of the type of stocking (flat or circular or micro-massaging) and the degree of compression, which can vary depending on the clinical stage, the shape of the limbs, the presence of edema, the symptoms, and the patient’s tolerance [1,2,3,4,7,8]. The therapeutic choice often becomes exclusively based on the doctor’s personal experience with patients, lacking a standardization of scientific evidence that can guide the choices of one treatment rather than another.

### 1.2. Pain, Adipocytes, and Compression

Pain is a leading and common complaint in lipedema. Patients find pain an extremely distressing symptom that affects the quality of life [9]. The pain is often described as pressure- or touch-induced, spontaneous, and sometimes described as more of a burning, tingling, and numbing sensation. It is becoming increasingly accepted by the scientific community that the therapeutic management of lipedema should consider the severity and location of pain present with the severity of the disease [10]. The causes of pain in lipedema are still being studied, but the postulated hypotheses are different. A first hypothesis is based on insufficient oxygen supply in the lipedema tissue: this would trigger an inflammatory reaction resulting in sprouting of fragile blood vessels and fibrosis. Lack of oxygen would also be the cause of adipocyte death. This hypothesis has been demonstrated by antibody tests: in regions where oxygen is lacking, there is an increase in phagocytes, which are part of the body’s innate immune response and may also be indicative of an inflammatory reaction. More studies suggest the presence of a chronic and insidious inflammatory process in lipedema. Other authors hypothesize that excessive fat accumulation within fat cells may induce a stress response that causes the release of proinflammatory factors from fat cells and other immune cells in the adipose tissue. Whatever the underlying process, pain in lipedema would appear to be mediated by nerve irritation triggered by inflammatory processes [11]. Indeed, the characteristics of pain that patients commonly report may be attributable to allodynia (nociceptive sensitization, in which a harmless stimulus is perceived as painful) and neuropathic pain [12,13]. The modulation of inflammation may play a key role in the pathogenesis of lipedema. The consequences of inflammation and the stagnation of interstitial fluid can result in the activation of nerve fibers, which may subsequently lead to the development of painful lipedema of adipose tissue [14]. In this regard, a reduced amplitude of the action potential in nerve conduction has been observed, attributable both to mechanical forces (edema, and above all, the increased adipose tissue) and to biochemical factors, such as inflammation of the sensory nerves caused by the sympathetic nerves that innervate adipocytes. The same authors argue that estrogens also have a central role in the regional sympathetic innervation of the SAT, which would lead to abnormalities in the innervation and concomitant inflammation of the sensory nerves.

Microangiopathy could also contribute to aggravating the already abnormal innervation, which would reduce the supply of nutrients to the nerves [15]. Chakraborty et al. identified alterations in neuronal density or indications of neurogenic inflammation that corroborate the underlying biological changes and potential inflammation in the lipedema tissue. Additionally, they observed that the expression of two neuropeptides (CGRP and NGF) that have previously been associated with nociceptive pain pathways or neurogenic inflammation is significantly altered in the skin of lipedema patients [16]. In conclusion, it is conceivable that to control pain in lipedema, it is useful to reduce inflammation, consequently reducing the irritation of the nerve fibers and the mechanism of neuropathic pain. A way to obtain this result could be compression therapy. The objective of compression therapy is to enhance venous and lymphatic flow, minimize capillary filtration, and provide support to the tissues, thereby improving the shape of the limbs. Additionally, compression therapy improves mobility, increases functionality, and facilitates movement, while also addressing other complicating factors, such as inflammation, cellulitis, and adipogenic changes. Due to its anti-inflammatory effect, it can reduce pain [17]. It should be noted that though this aspect is not universally accepted among lipedema experts, there is evidence that peripheral tissue lipid transport and homeostasis may be influenced by lymphatic function. This could explain the increased fat deposition observed in patients with lymphedema. Recent research suggests that the lymphatic role in lipid transport is an active and complex process. When lymphatic function is reduced or compromised, there are systemic consequences for lipid metabolism and transport. Given that the lymphatic system is responsible for returning interstitial proteins to the blood, it seems reasonable to conclude that extravascular lipoproteins would follow a similar principle. Though impaired lymph drainage may influence fat disposition and obesity, the reverse appears to be the case [18,19,20]. Another noteworthy topic is the phenomenon of mechanotransduction. A comprehensive review of the experimental studies addressing mechanotransduction in adipocytes and of computational and mathematical models useful for studying mechanotransduction and for quantifying the responsiveness of adipocytes to different types of mechanical loading reveals that adipogenesis is influenced by mechanical stimulations. It also demonstrates that different static or dynamic modes could have different effects on adipogenesis. In particular, static loads appeared to have a dual effect on adipogenesis. Static stretching was found to accelerate differentiation, whereas static compression was found to impede it. It was also found that multiple signaling pathways were involved in this process [21]. The effects of mechanical stimuli on adipogenesis were also investigated in animal models. As observed in vitro, dynamic loading delivered to adipose tissues in animals has been found to suppress adipogenesis and reduce body fat. The influence of mechanical loading on human adipose tissue in vivo was also characterized in several studies. These studies demonstrated that dynamic loading, such as mechanical massage or whole-body vibration, once more emerges as a loading mode that suppresses adipogenesis or even breaks down adipose tissues in vivo [22,23,24]. Hossain et al. investigated the effect of compressive force applied to a human preadipocyte cell line (Simpson–Golabi–Behmel Syndrome) before and after the start of adipogenesis induction, evaluating the levels of gene expression and the accumulation of lipids. In SGBS cells subjected to a compressive force of 226 Pa for 12 h before adipogenic in-duction, adipogenesis was inhibited, and compressive force immediately after adipogenic induction did not affect the adipogenesis. The compressive force inhibited adipogene-sis by suppressing expression of peroxisome proliferator-activated receptor (PPARγ2) and CCAAT/enhancer binding protein (C/EBP) in a cyclooxygenase-2 (COX-2)-dependent manner [25]. In another study, the effect of mechanical stimulation by compressive force on adipogenesis was investigated in adipose stem cells (ASCs): the authors found that mechanical compressive force reduced numbers of oil droplet-filled cells and downregulated mRNA levels of both PPARγ1 and adiponectin and protein levels of PPAR-γ, concluding that in culture medium containing adipogenic stimuli, mechanical compressive force inhibited adipogenesis of ASCs [26]. It is increasingly recognized that the interaction of adipose cells with extracellular mechanophysical environments may play an important role in regulating adipogenesis and the function of differentiated adipocytes, and this interaction may be mediated by adipose cell mechanics [27]. The new understanding that adipogenesis is influenced by mechanical stimulation has the potential to open important new research avenues, driven by mechanotransduction, to help us understand pathogenic mechanisms underlying not only obesity but also other rare adipose tissue diseases or lipedema. Furthermore, these studies could also be fundamental to guide us in the search for new therapeutic approaches based on mechanotransduction, such as, for example, in the case of elastic compression with micromassaging fabric. Through the micromassage fabric, it is possible to recreate eccentric and concentric stimuli on the superficial tissues to which it is applied, generating a biomechanical action based on alternating “compressive” and “decompressive” forces. The eccentric mode recreates an increase in skin and subcutaneous elasticity by exerting a “decompressive” action. It is assumed that a decompressive stimulus can reduce subcutaneous compression by contributing to the dilation of the interstitial spaces and to an increase in microcirculatory blood flow and the draining activity of the superficial lymphatic system. The concentric force produces a compressive stimulus by increasing the pressure gradient on the tissue to which it is applied, promoting decongestion towards the tissue subjected to decompressive stimuli. The aforementioned forces add to the movement to facilitate the reabsorption of the flows, creating a close relationship between micromassage fabric, skin and subcutaneous tissue. The objective of applying this type of texture is therefore to improve skin elasticity and blood and lymphatic microcirculation, obtaining an increase in drainage and microcirculatory activity [28]. Given these premises, it becomes simple to hypothesize that these effects could have a positive effect on lipedema tissue. The objective of this study was to investigate and present the clinical results obtained on patients who were treated with elastocompressive leggings with micromassage fabric associated with moderate physical activity and movement carried out in simple daily activities.

## 2. Materials and Methods

### 2.1. Population

This is a spontaneous observational study. We selected from patients with lipedema a group of females affected by columnar Type 3 lipedema, in Stages 1, 2, and 3, in reproductive age, who had never undergone decongestive treatments or had never worn elastic medical compression, had never undergone liposuction surgery, were not following a diet, and had not had any variations in weight > 10% in the last 6 months. Exclusion criteria were chronic diseases and any uncompensated endocrinological disorders such as type 1 and 2 diabetes mellitus or hypothyroidism, obesity caused by endocrine diseases or drug-induced obesity, and any estro-progestin therapy up to 2 months.

We included in this study 29 patients with these clinical characteristics and who had worn elastocompressive leggings with micromassage fabric (BE YOU TONIC, SOLIDEA^®^) for 4 weeks, a treatment that we generally recommend to all patients, from the moment of diagnosis, while waiting to complete any in-depth testing that we would require. Patients had to wear the leggings for three consecutive hours a day. During this period, they were asked to engage in moderate physical activity. This consisted of continuous walking for one hour, followed by two hours of usual daily activities. It was imperative that patients refrained from wearing leggings while lying down for a long time or if they were experiencing prolonged periods of inactivity. We asked them to write down every day in a diary the hours they wore the leggings.

We obtained informed consent from all patients recruited into the study to use the collected data.

The diagnosis of lipedema was based on the clinical findings of the characteristic symptoms and signs of the disease, as previously described in our previous work [29]. The clinical examination entailed a comprehensive evaluation of the entire body, with a focus on identifying characteristics that could inform diagnosis, clinical staging, and phenotyping. This included an assessment of the distribution and characteristics of lipedema tissue, as well as the appearance and conformation of the lower and upper limbs, abdomen, and back. Additionally, the examination encompassed an evaluation of the subcutis and skin, including the identification and characterization of subcutaneous nodulations, as well as an assessment of tissue laxity, signs of edema, and endocrinological and venous diseases.

### 2.2. Subjective Symptomatology

All patients were asked to answer the questions of a questionnaire to evaluate the presence of and changes in subjective symptoms. A questionnaire was administered to all patients consisting of 17 questions (ending with a final score) relating to the evaluation of the subjective symptoms present at the time of diagnosis (T0) and after 4 weeks of treatment (T1). The questions pointed out, in addition to pain, the presence of felling or swelling in the lower limbs, in the ankle, foot, and toes, and in other locations (for example, on the hands, around the eyes, etc.), feeling of heaviness, cold skin, hypersensitivity, paresthesia, burning and easy bruising on the lower limbs, easy tiredness, pain and swelling in the lower limbs during physical activity, and finally, pain and swelling in the lower limbs when standing for a long time. To quantify the extent of the symptoms, we used the Verbal Rating Scale (VRS) with a 6-point Likert-type scale with the following verbal descriptors: 0 = none, 1 = very mild, 2 = mild, 3 = moderate, 4 = strong, 5 = very strong [30]. Finally, a total symptom score was calculated, obtained from the average of the scores of the individual questions.

### 2.3. Clinical Assessment

**Figure 1:**
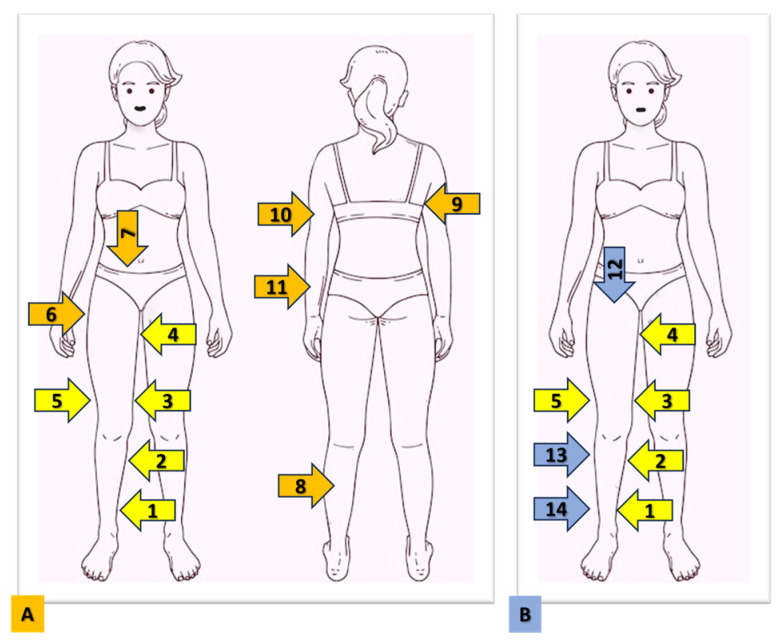
The figure shows the points where pain is detected with the Progressive Pain Check (PPC) method (**A**) and the points where ultrasound mixing of the thickness of the skin and adipose tissue is carried out (**B**). The first 5 points (from 1 to 5) are the same. The Lower Body Pain Score is calculated from the sum of point 1 to point 8. The Upper Body Pain Score is calculated from the sum of points 9, 10, and 11. The Total Body Pain Score or Ricolfi–Patton Score (RPS) is calculated with the sum of all points from 1 to 11. 1: medial lower third of the leg; 2: medial upper third of the leg; 3: medial lower third of the thigh; 4: medial upper third of the thigh; 5: later lower third of the thigh; 6: lateral upper third of the thigh 7: lower abdomen; 8: posterior middle third of the leg; 9: lateral edge of the tissue covering the teres major muscle; 10: arm; 11: forearm; 12: anterior upper third of the thigh; 13: lateral upper third of the leg; 14: lateral upper and lower third of the leg.

At the time of diagnosis (T0) and after 4 weeks (T1), we measured anthropometric parameters (weight, height, waist and hip circumference) and assessed the presence of clinically detected pain with tissue folding. We systematically applied the method we have called the Progressive Pain Check (PPC) at every medical visit in patients suffering from lipedema to monitor the disease. The method is not limited to the evaluation of the lower limbs alone or to the areas that seem most affected by the disease but always involves a complete evaluation of the lower part of the body (lower limbs and lower abdomen) and upper part of the body (back and upper limbs) of the body (Figure 1). Pain is quantified with the VRS, which is represented by a 5-point Likert-type scale with 5 verbal descriptors: no pain, mild pain, moderate pain, severe pain, and very strong pain [31]. The VRS was chosen because it is preferred by patients for simplicity compared to the Visual Analogue Scale (VAS) and Numerical Rating Scale (NRS) and considered more realistic for the definition of pain perceived [32,33].

For the lower part of the body, we evaluated the presence of pain in 8 points:

1. Medial lower third of the leg (about 5 cm above the medial malleolus);
2. Posterior middle third of the leg (immediately below the end of the gastrocnemius muscle);
3. Medial upper third of the leg (medial to the prominence of the tibial tuberosity);
4. Medial lower third of the thigh (at this point, the pain is assessed by applying a pressure on the tissue present in the popliteusspace; to identify it, we choose the tendons of the most medial thigh muscle as a reference point and pressed the tissue between the 4 fingers and the palm of the hand, not between the index and the thumb, as in the other points);
5. Medial upper third of the thigh (about 5 cm below the inguinal crease);
6. Lateral upper third of the thigh (immediately below the trochanter);
7. Lateral lower third of the thigh (about 5 cm above and lateral to the top of the patella);
8. Lower abdomen (under an imaginary line subtended between the upper apex of the pelvic bones and a point approximately 3 fingers below the navel).

For compression at these points, a pinch was applied to the tissue between the index finger and the thumb, with the exception of the point in the medial lower third of the thigh (as described above).

For the upper part of the body, we evaluated the presence of pain in 3 points:

1. Lateral edge of the tissue covering the teres major muscle;
2. Arm;
3. Forearm.

For the upper body, the examination was started behind the patient: the first point was evaluated by pinching the tissue between the thumb and index on the lateral edge of the tissue covering the teres major muscle. Then, standing in front of the patient, we began the examination by lifting the arm and pinching the tissue of the forearm in the proximal ulnar region, and finally, with the arm flexed 90° on the forearm, we evaluated the tissue under the bicipital groove.

To quantify the extent of the pain, we assigned a numerical value to the VRS items, from 0 to 4: 0 = no pain, 1 = mild pain, 2 = moderate pain, 3 = severe pain, 4 = very severe pain. From these data, we calculated 3 scores, derived from the mathematical sum of the values of each point included in the score:

- Lower Body Pain Score (LBPS): sum of the 8 points described above for the lower part of the body, which includes the limbs and abdomen (the score is defined as a value between 0 and 32);
- Upper Body Pain Score (UBPS): sum of the 3 points described above for the upper part of the body, which includes the back and upper limbs (the score is defined as a value between 0 and 12;
- Total Body Pain Score (TBPS): calculated with the sum of all 11 points evaluated, which we called Ricolfi–Patton Score (RPS). The RPS is defined as a value between 0 and 44.

### 2.4. Leg Volumes

Leg volumes were measured using the classical Kuhnke’s disc method [34], and circumferences were obtained at 4 cm intervals beginning at the foot and ending at the highest point of the inner thigh. In addition to the circumferences of the lower limb, 2 circumferences of the right and left feet were measured, respectively, at 4 and 8 cm from the nail base of the 5th finger.

### 2.5. Ultrasound Measurement

**Figure 2:**
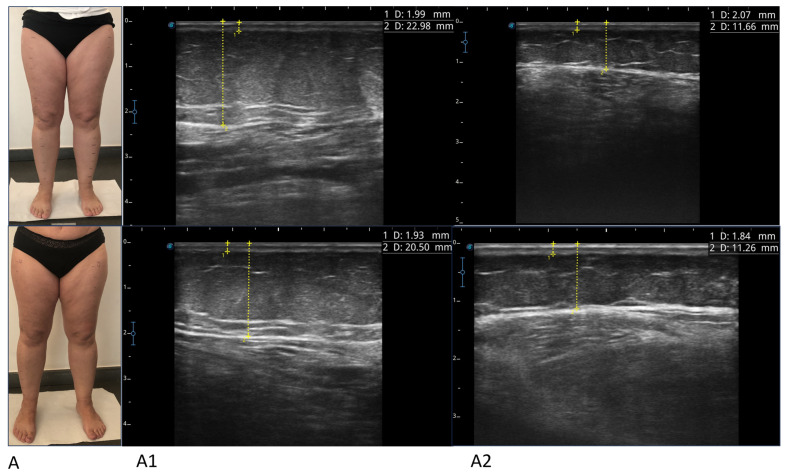

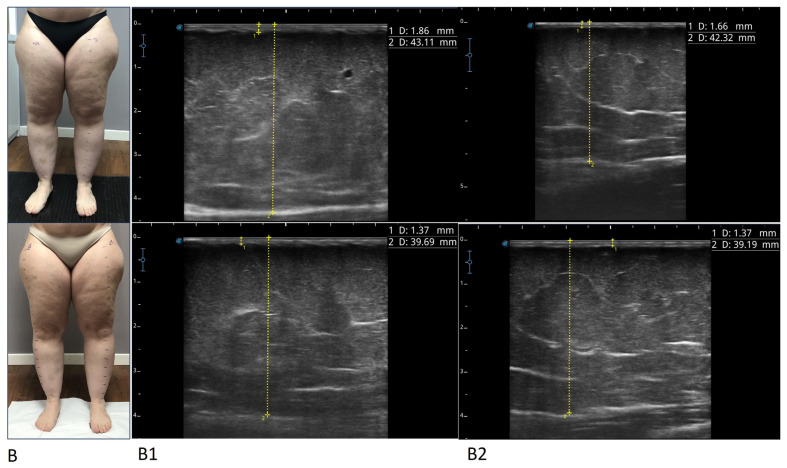
Photographs and ultrasounds of two patients (**A**,**B**), before treatment (images above) and after treatment (images below). The ultrasound measurements are represented by the dashed yellow lines: the thickness of the skin was measured from the skin surface to the lower edge of the epidermis and dermis complex, and the thickness of the suprafascial superficial adipose tissue was measured from the skin surface to the subcutaneous transition structure (fascia). All imaging was performed using a high-frequency linear probe (8–14 MHz), keeping the probe perpendicular to the skin and always without applying pressure. For patient A, ultrasound images relating to the lower medial third of the leg (**A1**) and the upper lateral third of the leg (**A2**) before and after the treatment are shown (box above and box below, respectively). For patient B, ultrasound images relating to the lower medial third of the thigh (**B1**) and the upper medial third of the thigh (**B2**) before and after the treatment are shown (box above and box below, respectively).

Ultrasound measurement of the adipose tissue of the lower extremities was performed. The measurement was conducted using a high-frequency linear probe with a frequency range of 8–14 MHz (SonoScape X3, SonoScape Medical Corp., Shenzhen, China). To ensure accuracy and minimize discomfort, the probe was maintained in a perpendicular position relative to the skin, and no pressure was applied to the underlying tissue. For each point, the following measurements were taken: the thickness of the skin, from the surface to the lower edge of the epidermis and dermis complex; and the thickness of the supra-fascial superficial adipose tissue, from the surface of the skin to the muscle fascia. The measurements were performed at 8 different points on the right and left lower limbs (Figure 2):

- Medial and lateral lower third of the leg (about 5 cm above the medial malleolus);
- Medial and lateral upper third of the leg (about 5 cm below the medial to the prominence of the tibial tuberosity);
- Medial and lateral lower third of the thigh (about 5 cm above the upper edge of the patella);
- Medial upper third of the thigh (about 5 cm below the inguinal crease);
- Anterior third of the thigh (about 5 cm below the inguinal crease).

The ultrasound examination was always performed with the patient lying supine and to the patient’s right. The evaluation begins from the lower medial third of the leg, proceeds with the evaluation of the points indicated on the medial side towards the groin, then continues distally along the lateral side of the limb up to the lower lateral third of the leg.

### 2.6. Statistical Analysis

Demographic and clinical data were summarized using descriptive statistics, measures of variability, and precision, depending on the type of variables (continuous or categorical). Skewness and a kurtosis test were performed to check the normality in the distribution of continuous variables. A two-sample paired *t* test (for normally distributed data) or its corresponding Wilcoxon matched-pairs signed-rank test (for non-normally distributed data) was performed to compare the means of body mass index (BMI), weight, waist, hip and limb circumferences, pain, symptoms, adipose tissue and skin thickness, and foot circumference and volumes as measured at baseline (T0) and after 4 weeks (T1). The same statistical tests were used to compare the means between the right and left side for volumes, adipose tissue, and skin thickness (T0 right vs. T0 left; T1 right vs. T1 left) and delta calculated as T0–T1 (delta T0 T1 right vs. delta T0 T1 left).

Three separate multivariate linear regression models were carried out to explore the relationship between volume of the right lower limbs T1, volume of left lower limbs T1, and Lower Body Pain Score T1, each taken individually as a dependent variable; models were adjusted for the same set of predictors (time of use for leggings, stage of disease, age, duration of disease, BMI, and the dependent variable measured at baseline).

Total Symptom Score was considered to be a dependent variable that was modeled by bounded multivariate linear regression (tobit: lower limit = 0, upper limit = 5) as adjusted for the same predictors previously listed.

Stata 17.0 SE-Standard Edition (StataCorp LLC, College Station, TX, USA) software was used to perform the statistical analysis.

## 3. Results

### 3.1. General Description, Phenotypic, and Staging

The population selected for this study was composed of 29 female patients. All patients were affected by columnar Type 3 lipedema, in different clinical stages of presentation: Stage 1 in 44.8%, Stage 2 in 27.6%, and Stage 3 in 27.6% of cases. A total of 72.4% of patients also had upper extremity involvement. The mean age of the patients was 37.9 (6.74) years. The mean and standard deviation (SD) of BMI was 28.9 (4.64) kg/m^2^, with a prevalence of obesity (BMI > 30 kg/m^2^) of 37.9%. The mean (SD) age of onset was 14.1 (9.55) years, and the mean (SD) of the disease duration at the time of our evaluation was of 23.8 (11.4) years. The mean (SD) time of wearing the garments was 3.7 (2.12) h (median of 3; IQR = 0.963). Clinical characteristics are shown in Table 1. There are no significant differences in age (*p* = 0.346), age of onset (*p* = 0.778), or in the duration of the disease (*p* = 0.772) between the stages (*p* = 0.346), whereas there is, as expected, a significant difference in BMI, with a progressive increase that increases with the severity of the clinical stage (*p* < 0.001). Family history of lipedema was investigated in first-, second-, and third-degree relatives: a positive family history was found in 75.9% of cases. Of these 29 patients, 20 did not take any drug therapy, and 9 patients assumed a chronic drug therapy: 4 patients were on levothyroxine therapy (for hypothyroidism), 1 patient with lamotrigine (for epilepsy), another patient assumed lamotrigine in association with aripiprazole in metilfenididate chlorhydrate (for Attention Deficit Hyperactivity Disorder and fibromyalgia), a patient assumed escitalopram (for depression), and another gabapentin (for anxiety); finally, another patient assumed ramipril (for arterial hypertension), and the last patient assumed salicylic acetyl acid for homozygous mutation of the V Leyden factor. 

No other therapy was assumed, No one assumed hormonal treatment, cortisone, Non-Steroidal Anti-Inflammatory Drugs (NSAIDs), or other anti-inflammatory and pain-relieving drugs. No patient had diabetes or severe or unstabilized chronic diseases. In patients taking levothyroxine, the adequacy of therapy with TSH dosage was confirmed.

In 41% of cases, estrogen-progestin therapy had been taken in the past for an average (SD) duration of 3.05 (3.77) years.

### 3.2. Anthropometric Measurements, Volumes, and Pain

Table 2 shows the change in anthropometric measurements, limb volumes, circumferences of the feet, and pain. There was no significant change in BMI, whereas a significant reduction in waist circumference (*p* = 0.040) and in hip circumference (*p* = 0.003) was observed. After four weeks of wearing the leggings, we observed that the skin appeared smoother, and that upon palpation, the tissues appeared more compact, and the temperature of the skin of the lower limbs became more uniform compared to the beginning of the study. Furthermore, we did not observe the appearance of pitting edema (particularly on the dorsum of the foot and toes) or the appearance of skin lesions and/or bruising following the use of the leggings. The volume of the lower limbs (both right and left) was significantly reduced after 4 weeks of treatment (*p* < 0.001 for both). The average volume reduction achieved was 7.10%. With the use of leggings, we did not detect any swelling in the foot; on the contrary, both the circumferences of the foot, on the right and on the left, were significantly reduced (Table 2). After 4 weeks of treatment, the pain evoked with the fold of the SAT of the areas affected by lipedema treated with the leggings was markedly reduced compared to the beginning of the study, as revealed by the reduction in the scores of Lower Body Pain Score and the Total Body Pain Score, also called the Ricolfi–Patton Score (RPS) (*p* < 0.001 for both scores). On the contrary, in the untreated areas, the evoked pain would seem to increase, as shown by the significant increase in the Upper Body Pain Score (*p* = 0.026).

### 3.3. Symptoms

Table 3 reports the variation in the extent of spontaneous pain and the different aspects relating to the characteristic symptoms of patients suffering from lipedema: after 4 weeks of treatment, an improvement in all these subjective symptoms was detected. 

The total symptom score was also significantly reduced (*p* < 0.001).

### 3.4. Ultrasound Measurement

As shown in Table 4, the thickness of the subcutaneous tissue measured with ultrasound was significantly reduced in all points of the lower limb evaluated on both the right and left, except for the medial and lateral lower third of the left thigh. 

The thickness of the skin measured in the same locations and both on the right and left was also significantly reduced in all points (Table 4). 

### 3.5. Other Considerations

#### 3.5.1. Comparison between Right and Left Limbs

The data relating to the volume of the limbs and the ultrasound measurements carried out between the right and left limbs were compared, as reported in Table 4. The data were compared at the beginning of the study and after the treatment, and a comparison was also performed of the amount of variation between the two sides. Before treatment, a significant difference in the volume calculated in the lower limbs was detected, with a prevalence of the volume of the right lower limb (*p* < 0.001). The symmetry of the lower limbs after treatment improved, and the difference between right and left was no longer significant (*p* = 0.279). The data relating to the comparison of the ultrasound measurements of the adipose tissue between right and left was, however, more inconsistent: from a statistical point of view, the thickness of the adipose tissue was greater on the right at four points (in the lower medial third of the leg, in the upper medial third of the thigh, and in the lower medial and lateral third of the thigh), and it was greater at a single point on the left (in the upper medial third of the leg). After the treatment, the points where the difference was significant were reduced, with a prevalence always only on the right (See Table 5). Evaluation of the difference in the variation in thicknesses detected in the different points between right and left did not show significant differences, with the exception of the lower medial third of the leg (*p* = 0.039).

#### 3.5.2. Multivariate Analysis of Factors Potentially Influencing Response to Treatment

Table 6 shows the results of the multivariate regression analysis that was carried out to evaluate the impact of the treatment on the volume of the lower limbs (right and left, Model 1 and 2), on pain (Lower Body Pain Score, Model 3), and on symptoms (Total Symptom Score, Model 4) adjusted for potential confounders (baseline variables, time of wearing leggings, age, BMI, duration of the disease, and stage of the disease). Models 1, 2, and 3, relating to lower limb volumes and pain, show similar results, showing a statistically significant link only with the respective variable measured at baseline, adjusted for all other predictors. The final effect based on these data depends only on the starting value of the same variable and not on the others (for details, see Table 6). In Model 4, relating to the Total Symptom Score, none of the factors considered, including the score value before treatment, showed a statistically significant effect. However, in this case, considering the small number of patients studied, the effect of the clinical stage has a clinically significant value (although not statistically significant): subjects with Stage 3, at the end of treatment, achieved a lower average of Total Score Symptoms than subjects in Stage 1 (coef. = −0.83, 95% CI = −1.74–0.079, *p*-value = 0.071). 

The same multivariate regression analysis was carried out to evaluate the impact of the treatment on the thickness of the adipose tissue and skin measured with ultrasound, using the same potential confounders (baseline variables, time of wearing leggings, age, BMI, duration of the disease, and stage of the disease).

On the right side, the thickness of the adipose tissue obtained after the treatment is statistically correlated in all points, except for the upper anterior third on the thigh with the initial thickness. The improvement of this parameter, understood as a reduction in the thickness of the adipose tissue, is not limited by any of the factors considered possibly interfering, except for the BMI, which negatively influences the outcome in two points of the limb: the upper anterior third of the thigh and the medial upper third of the thigh.

On the left side, the thickness of the adipose tissue obtained after the treatment is statistically correlated in all points, except for the medial upper third of the thigh with the initial thickness. The improvement of this parameter, understood as a reduction in the thickness of the adipose tissue, is not limited by any of the factors considered possibly interfering, with the exception of the BMI, which negatively influences the outcome in one point of the limb (upper anterior third of the thigh) and the duration of the disease, which negatively influences the outcome in another single point (lateral lower third of the thigh) (see Supplementary Materials, Tables S1 and S2).

As regards the thickness of the skin, the data are more uneven, and the analysis showed discordant data on the two limbs, for which it will be necessary to carry out further in-depth and confirmatory studies. To summarize the results, the factor with a statistically and or clinically significant impact on the thickness after the treatment is the initial thickness of the skin, though it does not appear to be influenced by the length of time the leggings have been worn, BMI, age, and duration of the disease (with the exception of the anterior upper third of the left thigh), nor by the clinical stage (with the exception of the medial upper third of the right leg).

## 4. Discussion

The aim of this work was to demonstrate that the use of elastic compression with micromassaging fabric in association with movement is effective in the multimodal treatment of lipedema patients and to propose clinical and instrumental methods useful for measuring these results, and therefore for the follow-up of the patients. In the literature, there are not many studies that demonstrate the effectiveness of conservative treatments in patients with this disease, and the methods used to evaluate the effects are manifold, not homogeneous, and hardly comparable. Before this publication, no other study was published on this type of device. 

Examining the literature, using the search term “lipedema” on Pubmed (until April 2024), for example, we found 17 studies (excluding case reports and studies that evaluated the effect of diet alone) that analyzed the effectiveness of different decongestive treatments, use of compression garments, and/or physical therapies in patients suffering from lipedema. As mentioned, different evaluation methods and different clinical parameters have been used. One of these parameters was pain, which was assessed using different scales: the most commonly used scales in these works were the VAS, with values from 0 to 10 [35,36,37,38,39,40], and the NRS, with values from 0 to 10 [41,42]; in a single study, the VAS with values from 0 to 100 [43] and the 5-point Likert scale were used [44]. In some works, multiple methods are used for pain assessment, such as the NRS 0-10, associated with the Wong Baker Faces Scale [45], or the combination of questionnaires with specific questions for the characterization of pain (with 4-point Likert scales) with a special numerical analog scale from 0 to 10 called the Pain Rating Scale (PRS) and the Wong Baker Faces Scale [46].

The first aspect to consider is that not only are different scales used, but the pain has been assessed generally as a spontaneous subjective or touch symptom, and an evaluation method has not yet been standardized for the systematic and objective clinical detection of the same. The second aspect is that excluding the studies reporting case reports, at the moment, a reduction in pain with the use of compression garments has been demonstrated in only two studies: the first was carried out with an online survey of 229 patients, and the second one is a clinical study that evaluated the effect of the use of a compression garment with exercise on pain; however, it was conducted on only three patients with lipedema [36,44].

We want to underline this aspect, because in our experience, the effect of this kind of treatment on pain, both spontaneous and provoked, would seem to be one of the most evident and relevant aspects from a clinical and subjective point of view and cannot be overlooked by the healthcare professional.

A reduction in pain has been demonstrated with other treatments, such as complete decongestive therapy (CDT); intermittent pneumatic compression therapy (IPCT) or exercise [40]; rapidly cycling hypobaric pressure [38]; microcurrents of bio resonance and transdermal delivery of active principles [35]; a combination of ketogenic diet and carboxytherapy [43]; a combination of CDT, IPCT, and walk-training [46]; a combination of multimodal manual therapy, compression, exercise, and education interventions [36]; and very recently, with a combination of defocused and radial shock wave therapy with mesotherapy and kinesio taping [42]. Partial results were obtained with a pneumatic compression device associated with a stocking in association with instruction for self-manual drainage, diaphragmatic breathing to assist lymph drainage, and skin care [45], and it was demonstrated that with subcutaneous adipose tissue therapy, the average daily pain improved significantly [41]. However, this result was not confirmed in the patients that had the same treatment after 4.5 months, maybe because the baseline pain was not so different from the previous study’s end pain scores [39].

In our study, we evaluated both spontaneous pain and the pain perceived during a clinical evaluation in specific points of the lower limb, abdomen, back, and upper limbs. We examined the variation in spontaneous pain using a simple questionnaire that surveys the presence and extent of pain and the other subjective symptoms: the treatment resulted in a significant reduction not only in spontaneous pain but also in all other aspects of subjective symptoms, and even more interestingly, it was demonstrated that the improvement occurred independently of the severity of the initial symptoms and regardless of time of wearing leggings, age, BMI, duration of the disease, and stage of the disease. Indeed, subjects with Stage 3 were those who obtained the greatest results. 

As regards the clinical detection of pain, the method that we have proposed is based on the evaluation of the pain provoked by pinching the skin: this method is, in our opinion, simple to apply and helps to evaluate the severity of the disease during the medical evaluation for the purpose of diagnosis. It is also fundamental to evaluate the effectiveness of the treatments proposed to patients. 

It should also be underlined that based on our clinical experience, the reduction in the subjective symptomatology that is easily observed after a cycle of decongestion treatment, diet, and/or targeted physical activity does not always correspond to the reduction in or disappearance of the pain caused by the pinch of the subcutaneous tissues, of which the patient is hardly aware.

Both aspects, the reduction of subjective symptoms and clinically assessed pain, are important but could also help us differentiate the type of treatment most indicated for the patient or even the need, for example, to continue or not with the decongestive treatments in progress. And from our results, it is clear that the use of this type of leggings with micromassage fabric for just 3 h a day, continuously and daily, is able to dramatically reduce both.

In this regard, two important aspects should also be highlighted in relation to the reduction in pain: the reduction in pain depends only on the value of the initial pain and occurs regardless of clinical stage, time of wearing leggings, age, BMI, and duration of the disease. This is a very encouraging result, unlike what one might think for a disease that tends to worsen over time and with weight gain.

The second aspect is that the effectiveness of the treatment is certainly mediated by the local effect of the leggings on the diseased tissue, given that it was obtained only at the level of the lower limbs and abdomen, whereas it was not detected in the other untreated areas, such as the upper limbs.

Another parameter used to evaluate the effectiveness of the conservative treatments studied is the change in the size of the treated lower limb, by measuring the volume of the limb. In our study, we used the method already known for studying volume variations in lymphedema, mathematically calculating the volume using the values of the limb circumferences measured every 4 cm. Other studies have used the same method to evaluate the change in volume, and it is therefore possible to compare the results obtained with the different treatments. Reduction in the volume of the lower limbs has been demonstrated for complete decongestive therapy (CDT) alone or associated with intermittent pneumatic compression therapy and physical exercise, with a percentage of volume reduction obtained varying from 5 to 10% [40,46,47,48]. A reduction of 5–6% has also been demonstrated with physical exercise alone and physical exercise associated with intermittent pneumatic compression [40]. Higher values of volumetric reduction were instead reported with SAT therapy, which determined a volumetric reduction of 13 and 19% (left and right lower limb, respectively), and in a subsequent study in which the treatment was repeated, the reduction achieved was 7% [39,41]. The studies are very different, not only with respect to the treatments, but also regarding the number of subjects studied, the population studied, and the duration of the study. However, it can be concluded that the reduction we obtained in our study with the use of leggings associated with physical activity allowed us to obtain a greater reduction in volume than that demonstrated for CDT alone or associated with IPCT with physical exercise or for physical exercise alone or associated with IPTC. The volumetric reduction, however, would appear to be smaller than the association of CDT+ IPTC and SAT therapy.

Considering that this is the first time that a volume reduction has been demonstrated with this method and with the use of leggings, and that this treatment does not require the use of machinery and/or the application of bandages or treatments by highly specialized healthcare personnel, it should be stressed that this is a treatment that the patient can carry out independently, saving work, time, and money.

Finally, still regarding this aspect, it is fundamental in our opinion to underline how the result is obtained regardless of age, disease duration, BMI, disease stage, and the hours of wearing the leggings.

Ultrasound evaluation for detecting the effectiveness of a treatment in patients suffering from lipedema was used in four studies: in one study, it was used to evaluate fibrosis in the subcutaneous adipose tissue in the lower abdomen and in the thigh [41]; in the other study, modification of the fat tissue structure (number and dimension of hyperechoic masses, visualization of the fascia and fluid in the tissue) was evaluated before and after therapy on the leg, thigh, and calf [39].

In the last two studies, which are more recent than the others, and both pilot studies, ultrasound measurement of the SAT was carried out in different points of the treated limbs. In the first study, the thickness of the skin and adipose tissue at four certain reference points was measured (half the length in the front of the thigh, half the length in the front of the shin; half the length on the side of the shin; above the medial malleolus) in two treatment groups, one group undergoing an exercise program and the other group undergoing an exercise program combined with flat-knitted class 2 compression leggings for 4 and 6 weeks. The thickness of the skin did not vary significantly between groups and did not change during the therapeutic process [36].

In the last interesting study, the authors evaluated using ultrasound to measure the thickness of the SAT in specific points of the thigh and leg (trochanteric region, proximal, middle, and distal thighs and legs) SAT echogenicity and elasticity were also measured [42]. Considering the measurement of the thickness of the subcutaneous tissue, a reduction was demonstrated in all points observed after treatment with defocused and radial shock wave therapy, mesotherapy, and kinesio taping. The measurement points and methods do not correspond to those used in our study.

The choice of the measurement points of the subcutaneous tissue, in our case, was made on the basis of our previous experience with this technique on patients with lymphedema and also for the simplicity of execution, with the patient lying supine.

This method, in our opinion, is simple to perform and can be used for the initial evaluation of the patient, both in quantitative and qualitative terms, and for follow-up.

However, it should be kept in mind that ultrasound is an operator-dependent method, and that, especially in the case of the lower limbs of patients with lipedema, where dysmorphisms in the shape of the limb are present, and the tissues are often very loose, which can result in significant discrepancies between measurements.

In this regard, among the objectives of this study, there was also that of comparing the measurements obtained between the right and left limb. We have found that there are significant differences between one side and the other; this could also be influenced by the different position of the operator with respect to the right and left limb. But the fundamental fact that emerged and that we want to underline is that to evaluate the effect of a conservative treatment applied equally bilaterally, as in our case, it may be sufficient to detect the variation on only one side, a choice that could also reduce the error depending on the operator. For simplicity of execution, considering the position of the doctor, who is generally on the right of the patient for clinical evaluation, we have also chosen the right side for the next studies.

Evaluating the result of the treatment with the ultrasound method, we demonstrated that the thickness of the SAT was significantly reduced in all points both on the right and on the left, except for the thickness measured at the level of the lower lateral and medial third of the left thigh (while being significantly reduced on the right). From a clinical point of view, this result is, in our opinion, very interesting for several reasons, not only because it is a demonstration of the treatment’s effect at this level, but also because the method, if compared with the measurement of the circumference, could be more specific. In fact, the circumference of the limb could be reduced for several reasons, not only due to a reduction in adipose tissue but also to a reduction in the thickness of the underlying muscle, for example. Furthermore, with ultrasound, the presence of edema with macroscopic lymphatic effusion can also be highlighted, a reduction in which would evidently also lead to a reduction in the measured circumference.

Ultrasound evaluation, in our opinion, should always be used, not only for diagnosis but also for follow-up.

With the ultrasound study, we also demonstrated that the treatment resulted in a significant reduction in the thickness of the skin at all points assessed. At the moment, though the thickness of the skin is an important element for the ultrasound evaluation of lymphedema, which characteristically presents a greater increased thickness, from the studies reported in the literature performed with ultrasound, the presence of a difference between the thickness or ultrasound representation of the skin is not detected in patients suffering from lipedema and controls [49,50,51]. However, in a biopsy study, increased epidermis thickness without increased fibrosis in lipedema was instead detected [52]. It is conceivable that this effect may be linked to an improvement in lymphatic drainage and/or a reduction in tissue inflammation, which is also suggested by the improvement in the symptoms and pain caused.

However, the limitation of this study remains the large sample size and the need to confirm the results obtained, although they are very promising.

A further limitation and an area for future investigation would be to ascertain whether the same outcomes can be achieved in males with lipedema. In this regard, it is known that there are leggings with the same micromassage fabric, designed specifically for men. However, during this study, there have been no cases of lipedema in male patients. Given that pain could arguably be a crucial diagnostic indicator also in males, and that one of the primary outcomes of this treatment is the alleviation of this symptom, it is plausible that the treatment may prove effective, at least in this regard. However, given the anatomical and structural diversity of adipose and subcutaneous tissues between men and women, the effect on the other parameters may be less obvious and needs to be evaluated with a targeted study.

## 5. Conclusions

The results obtained with the use of this type of elastocompressive garment with micromassage fabric in patients suffering from lipedema are encouraging from many points of view. The methods of use for patients are simple, do not require a great deal of effort, and are implemented autonomously, since they do not require the intervention of highly specialized healthcare personnel. It is sufficient to use the garment for 3 h a day, during which time it is advisable to carry out simple movement physical, which is also useful for improving other aspects of these patients’ symptoms. The leggings are easy to wear and do not cause discomfort. By following this simple protocol, patients reported a significant reduction in spontaneous pain, quantified according to the VRS, and at the same time, they also showed a reduction in provoked pain quantifiable from a clinical point of view with the methodology we have explained and proposed (the Progressive Pain Check). This is not a consistently achievable result, and it is parallel to the reduction in spontaneous pain, which adds value to this treatment. These data take on particular importance, as pain and discomfort are one of the main problems linked to this pathology and also affect the patient’s quality of life. Furthermore, the use of this device would also seem to allow these patients to carry out physical activity without worsening the symptoms, another aspect that has a particular impact on the lives of these patients, who are very often at a young age and want to commit to improving their health and their appearance or to simply carry out physical activity without limitations compared to one’s peers and healthy women. In our opinion, this type of treatment can be considered an integral part of conservative treatment, also because with these methods of use, we have seen that this garment, although not covering the foot, does not cause edema or discomfort in the foot in the patients studied but rather improves it, and it is therefore a garment that is not only useful but also more comfortable to wear during physical activity. To underline what has been said, the study has demonstrated the garment’s effectiveness in reducing the volume and thickness of adipose tissue in the ultrasound study; these are fundamental parameters that can be measured.

Finally, with the results obtained with this study, we propose a systematic and repeatable method that allows us to evaluate and quantify the pain and the volume and thickness of the adipose tissue and skin, parameters which, as we have demonstrated, can also be evaluated simply at the level of only one of the two limbs. These clinical and instrumental data are added to the evaluation of subjective symptoms and are fundamental aspects in our opinion. We hope they can become an integrative part of the evaluation of each patient at the time of diagnosis, for the patient’s follow-up, and to evaluate the effect of a treatment, whether conservative or surgical. 

## Figures and Tables

**Table 1:** Anthropometric and clinical data.

Variables	
Age (years)—mean (sd)	37.9 (6.74)
Age of onset (years)—mean (sd)	14.1 (9.55)
Duration of disease (years)—mean (sd)	23.8 (11.4)
Body mass index (kg/m^2^)—mean (sd)	28.9 (4.64)
Body weight (kg)—mean (sd)	78.2 (14.6)
Waist circumference (cm)—mean (sd)	92.3 (10.2)
Hip circunference (cm)—mean (sd)	117 (11.6)
Stage 1—n (%)	13 (44.8)
Stage 2—n (%)	8 (27.6)
Stage 3—n (%)	8 (27.6)
Upper limb involvement—n (%)	21 (72.4)
Time of use of garments (hours)—mean (sd)	3.7 (2.12)

Anthropometric and clinical data of the patients before starting the treatment.

**Table 2:** Anthropometric data, volumes, and pain.

Variables	T0	T1	*p* Value
Body weight (kg)	78.2 (14.6)	77.7 (14.9)	0.053
Waist circumference (cm)	92.3 (10.2)	91.1 (11.0)	0.040
Hip circumference (cm)	117 (11.6)	116 (12.0)	0.003
Right leg volume (mL)	14,974 (3024)	13,747 (3149)	<0.001
Left leg volume (mL)	14,449 (2766)	13,588 (2823)	<0.001
Right foot circumference 4 cm	22.0 (1.42)	21.7 (1.33)	0.020
Right foot circumference 8 cm	22.9 (1.12)	22.6 (1.06)	0.013
Left foot circumference 4 cm	22.0 (1.34)	21.4 (1.27)	<0.001
Left foot circumference 8 cm	22.8 (1.08)	22.4 (1.04)	<0.001
Lower Body Pain Score	3.03 (0.514)	0.560 (0.466)	<0.001 *
Upper Body Pain Score	1.83 (1.39)	2.00 (0.253)	0.026 *
Total Body Pain Score or Ricolfi–Patton Score	2.43 (0.870)	1.28 (0.776)	<0.001 *

Anthropometric data, values of the circumference of the feet measured at 4 and 8 cm from the base of the first toe, volume of the lower limbs, and pain scores caused by the adipose tissue fold before (T0) and after (T1) 4-week treatment. All data are expressed with mean (sd), including pain scores. Lower Body Pain Score: average of the eight points for the lower part of the body; Upper Body Pain Score: average of the three points for the upper part of the body; Total Body Pain Score: average of all points. The comparison analysis was performed with a paired *t* test, except for cases with non-normal distribution, for which the Wilcoxon test (*) was used.

**Table 3:** Symptoms.

Variables	T0	T1	*p* Value
Spontaneus pain in the lower limbs	3.10 (1.57)	1.21 (1.24)	<0.001 *
Feeling of swelling in the lower limbs	3.97 (0.906)	1.66 (1.17)	<0.001
Feeling of heaviness in the lower limbs	3.86 (0.990)	1.52 (1.27)	<0.001
Sensation of swelling in the ankles	2.62 (1.68)	1.28 (1.03)	<0.001
Sensation of swelling in the foot (back and toes)	0.552 (0.948)	0.242 (0.095)	0.032 *
Sensation of swelling elsewhere (on the hands, around the eyes)	0.759 (1.06)	0.414 (0.733)	0.005 *
Skin cold to the touch on the lower limbs	2.59 (1.52)	1.45 (1.35)	<0.001
Hypersensitivity to touch in the lower limbs	2.76 (2.03)	1.10 (1.05)	<0.001 *
Pressure pain in the lower limbs	4.21 (1.11)	1.72 (1.13)	<0.001 *
Paresthesia and tingling in the lower limbs	1.66 (1.56)	0.621 (0.903)	<0.001 *
Burning sensation in the lower limbs	1.41 (1.55)	0.483 (1.31)	<0.001 *
Eeasy bruising	2.74 (1.58)	1.38 (1.24)	<0.001 *
Fatigue in the lower limbs when performing movement or physical activity	3.38 (1.74)	1.45 (1.24)	<0.001
Pain in the lower limbs during or after movement/physical activity	2.55 (1.80)	1.21 (1.21)	<0.001 *
Swelling or discomfort in the lower limbs following physical activity	2.55 (1.72)	1.72 (1.20)	<0.001 *
Pain in the lower limbs when standing for a long time	3.48 (1.21)	1.66 (1.26)	<0.001
Swelling/discomfort in the lower limbs when standing for a long time	3.69 (1.32)	1.86 (1.27)	<0.001
**Total Symptom Score**	2.71 (0.883)	1.20 (0.701)	<0.001

Values relating to the intensity of subjective symptoms at the beginning of the study (T0) and after 4 weeks of treatment (T1). The score to assess the extent of the symptoms assessed was the Verbal Rating Scale (VRS), with a 6-point Likert-type scale with the following verbal descriptors: 0 = none, 1 = very mild, 2 = mild, 3 = moderate, 4 = strong, 5 = very strong. The Total Symptom Score was calculated with the average of the 17 items. The data are expressed as mean (SD). The comparison analysis was performed with a paired *t* test, except for cases with non-normal distribution, for which the Wilcoxon test (*) was used.

**Table 4:** Ultrasound measurements.

Variables	Right Lower Limb			Left Lower Limb		
	T0	T1	*p* Value	T0	T1	*p* Value
Thickness of **Adipose tissue**						
Lower medial third of the leg (mm)	23.3 (6.78)	21.1 (6.40)	<0.001 *	22.2 (5.92)	20.8 (5.68)	<0.001
Medial upper third of the leg (mm)	23.9 (7.99)	22.0 (7.07)	0.007	25.5 (7.71)	22.5 (8.28)	<0.001 *
Medial lower third of the thigh (mm)	37.4 (9.64)	36.0 (10.6)	0.010	33.9 (8.66)	32.0 (8.82)	0.071
Medial upper third of the thigh (mm)	34.3 (9.68)	31.6 (9.06)	0.002	32.0 (8.15)	26.9 (7.55)	<0.001
Upper anterior third of the thigh (mm)	17.7 (6.85)	15.2 (5.52)	<0.001 *	18.7 (7.54)	16.6 (7.54)	0.005 *
Lateral lower third of the thigh (mm)	24.7 (10.7)	21.9 (7.69)	0.004 *	20.1 (7.59)	19.2 (7.10)	0.223 *
Upper lateral third of the leg (mm)	15.0 (4.74)	14.1 (5.47)	0.002 *	14.0 (4.69)	12.1 (4.08)	0.003 *
Lower lateral third of the leg (mm)	13.1 (5.94)	10.8 (5.23)	0.002	13.6 (4.29)	12.1 (4.39)	0.001
Thickness of **Skin**						
Lower medial third of the leg (mm)	1.95 (0.240)	1.79 (0.184)	0.005 *	1.94 (0.242)	1.77 (0.239)	0.005 *
Medial upper third of the leg (mm)	1.85 (0.258)	1.738 (0.241)	0.016	1.79 (0.297)	1.65 (0.210)	0.009 *
Medial lower third of the thigh (mm)	1.89 (0.290)	1.64 (0.236)	<0.001	1.81 (0.351)	1.61 (0.232)	0.003
Medial upper third of the thigh (mm)	1.83 (0.30)	1.69 (0.31)	0.013	1.81 (0.285)	1.62 (0.268)	0.008 *
Upper anterior third of the thigh (mm)	1.74 (0.268)	1.59 (0.243)	<0.001	2.02 (1.89)	1.51 (0.217)	0.007 *
Lateral lower third of the thigh (mm)	1.99 (0.284)	1.807 (0.219)	<0.001	2.03 (0.288)	1.82 (0.248)	<0.001
Upper lateral third of the leg (mm)	1.88 (0.197)	1.78 (0.224)	0.010	1.95 (0.249)	1.72 (0.193)	<0.001
Lower lateral third of the leg (mm)	1.84 (0.225)	1.72 (0.179)	0.004	1.80 (0.261)	1.67 (0.155)	0.002

Ultrasound measurements of the adipose tissue and skin of the right and left lower limb at the beginning of the study (T0) and after 4 weeks of treatment (T1). The data are expressed with mean (SD). The comparison analysis was performed with a paired *t* test, except for cases with non-normal distribution, for which the Wilcoxon test (*) was used.

**Table 5:** Comparison between the right and left side of the body.

Variables	T0			T1			Delta T0–T1		
	Right	Left	P T0	Right	Left	P T1	Right	Left	P Delta
Volume of lower limbs (mL)	14,974 (3024)	14,449 (2766)	<0.001	13,747 (3149)	13,588 (2823)	0.279	1227 (947)	861 (914)	0.015
Thickness of adipose tissue									
Lower medial third of the leg (mm)	23.3 (6.78)	22.2 (5.92)	0.037 *	21.1 (6.40)	20.8 (5.68)	0.538 *	2.13 (1.98)	1.36 (1.57)	0.039
Medial upper third of the leg (mm)	23.9 (7.99)	25.5 (7.71)	0.025	22.0 (7.07)	22.5 (8.28)	0.524 *	1.88 (0.388)	3.00 (3.47)	0.223
Medial lower third of the thigh (mm)	37.4 (9.64)	33.9 (8.66)	0.001	36.0 (10.6)	32.0 (8.82)	0.002	1.50 (3.29)	1.87 (6.64)	0.799
Medial upper third of the thigh (mm)	34.3 (9.68)	32.0 (8.15)	0.015	31.6 (9.06)	26.9 (7.55)	<0.001	2.66 (4.55)	5.13 (4.90)	0.107 *
Upper anterior third of the thigh (mm)	17.7 (6.85)	18.7 (7.54)	0.436 *	15.2 (5.52)	16.6 (7.54)	0.056 *	2.49 (3.38)	2.08 (4.26)	0.642 *
Lateral lower third of the thigh (mm)	24.7 (10.7)	20.1 (7.59)	0.004 *	21.9 (7.69)	19.2 (7.10)	0.020 *	2.81 (6.94)	0.84 (4.61)	0.239 *
Upper lateral third of the leg (mm)	15.0 (4.74)	14.0 (4.69)	0.122 *	14.1 (5.47)	12.1 (4.08)	0.006 *	0.897 (3.47)	1.85 (3.26)	0.482 *
Lower lateral third of the leg (mm)	13.1 (5.94)	13.6 (4.29)	0.472	10.8 (5.23)	12.1 (4.39)	0.098	2.26 (3.73)	1.47 (2.36)	0.285

Volume data and ultrasound measurements of the adipose tissue between right and left at baseline (T0) and after 4 weeks of treatment (T2) and the variation obtained with the treatment on the right (Delta T0–T2 right) and on the left (Delta T0–T2 Left). P T0 refers to the comparison between the right side and the left side at baseline. P T1 refers to the comparison between right and left side after treatment. P Delta refers to the contrast between the change to the right and left. The data are expressed with mean (SD). Statistical analysis was performed with a paired *t* test, except for cases with non-normal distribution, for which the Wilcoxon test (*) was used.

**Table 6:** Multivariate regression analysis.

Dependent Variable:	Volume Right Lower Limbs T1 Model 1			Volume Left Lower Limbs T1 Model 2			Lower Body Pain Score T1 Model 3			Total Symptom Score T1 Model 4		
	Coefficient	[95% Conf. Interval]	*p* Value	Coefficient	[95% Conf. Interval]	*p* Value	Coefficient	[95% Conf. Interval]	*p* Value	Coefficient	[95% Conf. Interval]	*p* Value
Dependent variable T0	1.012	0.786–1.24	0.000	1.05	0.807–1.29	0.000	0.521	0.070–0.972	0.026	0.133	−0.146–0.114	0.333
Time of use leggings	−27.0	−243–189	0.797	−39.9	−244–164	0.688	−0.042	−0.134–0.050	0.350	−0.100	−0.225–0.025	0.110
Stage 2	−93.8	−1213–1025	0.863	−220	−1284–843	0.671	0.249	−0.225–0.722	0.287	−.049	−0.695–0.596	0.876
Stage 3	−725	−2362–911	0.367	−1179	−2761–403	0.136	−0.083	−0.753–0.587	0.799	−0.832	−1.74–0.079	0.071
Age	−45.0	−126–35.8	0.260	6.86	−69.9–83.6	0.854	0.006	−0.230–0.041	0.740	−.026	−0.072–0.021	0.264
Duration of disease	27.7	−18.2–73.5	0.223	0.513	−42.9–43.9	0.981	0.004	−0.015–0.024	0.664	0.010	−0.017–0.036	0.449
BMI	59.2	−106–224	0.463	35.0	120–190	0.644	−0.031	−0.984–0.036	0.346	0.074	−0.008–0.158	0.076

Results of 4 distinct multivariate linear regression models in which the dependent variable measured after 4 weeks of treatment (T1) in Model 1 is the volume of the right lower limb; in Model 2, the volume of the left lower limb; in Model 3, the Lower Body Pain Score; and in Model 4, Total Symptom Score. For each model, the set of independent variables is the same (time use of leggings, clinical stage, age, duration of the disease, and BMI), and furthermore, for each model, the same dependent variable measured at time 0, before treatment, was added. BMI: body mass index.

## Data Availability

The derived data supporting the findings of this study are available from the corresponding authors upon reasonable request.

## Supplementary Materials

The following supporting information can be downloaded at: https://www.mdpi.com/article/10.3390/life14070854/s1, Table S1: Multivariate regression analysis, right lower limb, Table S2: Multivariate regression analysis, left lower limb.
