# Supplementary material for: Micromassage Compression Leggings Associated with Physical Exercise: Pilot Study and Example of Evaluation of the Clinical and Instrumental Effectiveness of Conservative Treatment in Lipedema
Source: Life (Basel). 2024 Jul 8;14(7):854. doi: 10.3390/life14070854 (PMC11278218; doi:10.3390/life14070854)
Supplement: Supplementary file 1 [file life-14-00854-s001.zip › life-3043588-supplementary.pdf]

Table S1. Multivariate regression analysis, right lower limb

The table shows the results of 8 distinct multivariate linear regression models in which the dependent variable measured after 4 weeks of treatment (T2) in in the listed models corresponds to the thickness of the adipose tissue measured in the specific points indicated, relating to the right lower limb.
For each model the set of independent variables is the same (time use of leggings, clinical stage, age, duration of the disease and BMI) and furthermore for each model the same dependent variable measured at time 0, before treatment, was added.
BMI: Body Mass Index
AT: adipose tissue

**RIGHT LOWER LIMB**	Dependent variable T0 (mm)	Time of use garments (hours)	Stage 2	Stage 3	Age (years)	Duration of disease (years)	BMI (kg/m$^{2)}$
**Model 1.** **Dependent variable:** thickness of AT T2, lower medial third of the leg							
**Coefficient**	.8867143	.0312054	-.5268217	-1.335235	.0384577	.0115278	.183934
**[95% conf. interval]**	.7398804 1.033548	-.3794412 .441852	-2.718092 1.664449	-4.669961 1.99949	-.1170851 .1940004	-.0772114 .1002669	-.0900053 .4578733
**P VALUE**	0.000	0.876	0.622	0.414	0.612	0.790	0.177
**Model 2.** **Dependent variable:** thickness of AT T2, medial upper third of the leg							
**Coefficient**	.7715472	-.3624822	3.072444	3.80268	.1772654	-.139567	-.2373593
**[95% conf. interval]**	.5770127 .9660817	-1.026884 .3019194	-.3460673 6.490956	-1.104939 8.710298	-.0744274 .4289582	-.2825897 .0034557	-.6954613 .2207427
**P VALUE**	0.000	0.269	0.076	0.122	0.158	0.055	0.293
**Model 3.** **Dependent variable:** thickness of AT T2, medial lower third of the thigh							
**Coefficient**	.9526676	.0113073	1.333486	1.137452	-.0956577	-.0661062	.1450165
**[95% conf. interval]**	.7432734 1.162062	-.7190028 .7416175	-2.523824 5.190797	-4.550894 6.825797	-.3646739 .1733586	-.2188681 .0866556	-.3489875 .6390206
**P VALUE**	0.000	0.975	0.480	0.682	0.468	0.378	0.548
**Model 4.** **Dependent variable:** thickness of AT T2, medial upper third of the thigh							
**Coefficient**	.3691074	.6549272	.8296575	.3839194	-.0187082	-.1333841	1.02374
**[95% conf. interval]**	.0643385 .6738764	-.1656094 1.475464	-3.373391 5.032706	-5.447931 6.21577	-.3111801 .2737638	-.3233152 .056547	.3728699 1.67461
**P VALUE**	0.020	0.112	0.686	0.892	0.895	0.159	0.004
**Model 5.** **Dependent variable:** thickness of AT T2, upper anterior third of the thigh							
**Coefficient**	.2506343	.0857114	-3.702652	-.3016745	-.0756342	.0381287	.7378755
**[95% conf. interval]**	-.052053 .5533217	-.3978335 .5692563	-6.385223 - 1.020082	-3.853378 3.250029	-.2633529 .1120845	-.0655043 .1417618	.2279455 1.247806
**P VALUE**	0.100	0.716	0.009	0.861	0.412	0.453	0.007
**Model 6.** **Dependent variable:** thickness of AT T2, lateral lower third of the thigh							
**Coefficient**	.5040458	-.6238231	4.290781	1.531807	.2256778	-.1312641	.0317755
**[95% conf. interval]**	.2288553 .7792363	-1.602012 .3543659	-.7848143 9.366376	-5.924662 8.988276	-.1458932 .5972488	-.3454592 .0829311	-.7019988 .7655499
**P VALUE**	0.001	0.199	0.093	0.674	0.220	0.216	0.929
**Model 7.** **Dependent variable:** thickness of AT T2, upper lateral third of the leg							
**Coefficient**	.7044704	.0162382	.3657872	1.404983	-.0340252	-.0005557	.3405623
**[95% conf. interval]**	.347786 1.061155	-.6905154 .7229918	-3.591648 4.323222	-4.489957 7.299923	-.2989137 .2308633	-.1540365 .152925	-.1301289 .8112535
**P VALUE**	0.001	0.962	0.849	0.625	0.792	0.994	0.147
**Model 8.** **Dependent variable:** thickness of AT T2, lower lateral third of the leg							
**Coefficient**	.5722481	.1555739	2.019657	5.075516	.2127818	-.1336866	-.242081
**[95% conf. interval]**	.3272879 .8172084	-.4957402 .8068879	-1.527307 5.566621	-.3845925 10.53563	-.0326161 .4581797	-.272579 .0052059	-.6916502 .2074881
**P VALUE**	0.000	0.625	0.250	0.067	0.086	0.058	0.275

Table S2. Multivariate regression analysis, left lower limb

The table shows the results of 8 distinct multivariate linear regression models in which the dependent variable measured after 4 weeks of treatment (T2) in in the listed models corresponds to the thickness of the adipose tissue measured in the specific points indicated, relating to the left lower limb.
For each model the set of independent variables is the same (time use of leggings, clinical stage, age, duration of the disease and BMI) and furthermore for each model the same dependent variable measured at time 0, before treatment, was added.
BMI: Body Mass Index, AT: adipose tissue

LEFT LOWER LIMB	Dependent variable T0 (mm)	Time of use garments (hours)	Stage 2	Stage 3	Age (years)	Duration of disease (years)	BMI (kg/m^{2})
**Model 1.** **Dependent variable:** thickness of AT T2, lower medial third of the leg							
**Coefficient**	.8934615	.233004	-.7534505	-.7262311	-.0410675	.0214195	.2109353
**[95% conf. interval]**	.7858803 1.001043	-.0545185 .5205266	-2.299203 .792302	-2.979582 1.527119	-.1489988 .0668637	-.0398968 .0827359	.0204504 .4014202
**P VALUE**	0.000	0.107	0.322	0.510	0.438	0.476	0.032
**Model 2.** **Dependent variable:** thickness of AT T2, medial upper third of the leg							
**Coefficient**	.8715301	.0290124	.1771978	1.804909	.0337859	-.0206044	.1365699
**[95% conf. interval]**	.6165075 1.126553	-.760241 .8182659	-3.879079 4.233474	-3.954457 7.564275	-.2626472 .330219	-.1922432 .1510343	-.433478 .7066178
**P VALUE**	0.000	0.940	0.928	0.522	0.815	0.805	0.624
**Model 3.** **Dependent variable:** thickness of AT T2, medial lower third of the thigh							
**Coefficient**	.4708685	-.1540627	4.340271	.4585533	-.1932233	.1050431	.7932822
**[95% conf. interval]**	.0600512 .8816859	-1.434167 1.126041	-2.251711 10.93225	-9.889193 10.8063	-.6625439 .2760974	-.1589262 .3690125	-.0286659 1.61523
**P VALUE**	0.027	0.805	0.185	0.927	0.402	0.417	0.058
**Model 4.** **Dependent variable:** thickness of AT T2, medial upper third of the thigh							
**Coefficient**	.4186955	.2117165	1.218493	4.074215	.1455069	-.053786	.3544904
**[95% conf. interval]**	-.0657995 .9031904	-.7831327 1.206566	-4.010704 6.447691	-3.160917 11.30935	-.208388 .4994019	-.2987006 .1911286	-.4423942 1.151375
**P VALUE**	0.087	0.663	0.633	0.255	0.402	0.653	0.365
**Model 5.** **Dependent variable:** thickness of AT T2, upper anterior third of the thigh							
**Coefficient**	.5435959	-.4345661	-2.955517	.2952678	.029845	.0255659	.6153056
**[95% conf. interval]**	.2462512 .8409407	-1.201747 .3326149	-6.916539 1.005506	-5.474778 6.065313	-.2603488 .3200388	-.1389219 .1900537	.0377489 1.192862
**P VALUE**	0.001	0.252	0.136	0.916	0.833	0.750	0.038
**Model 6.** **Dependent variable:** thickness of AT T2, lateral lower third of the thigh							
**Coefficient**	.5594951	-.1584413	.1605841	-.3350094	-.2351065	.2122718	.5677884
**[95% conf. interval]**	.2664981 .8524922	-1.007761 .6908781	-4.077387 4.398555	-6.387646 5.717627	-.5419216 .0717085	.0392732 .3852703	-.0139643 1.149541
**P VALUE**	0.001	0.702	0.938	0.909	0.126	0.019	0.055
**Model 7.** **Dependent variable:** thickness of AT T2, upper lateral third of the leg							
**Coefficient**	.5742134	.1176057	-.5609833	1.968193	-.0596505	.0244868	.0455059
**[95% conf. interval]**	.3033269 .8450998	-.485814 .7210253	-3.809962 2.687995	-2.657774 6.59416	-.2881138 .1688129	-.1047446 .1537183	-.3577142 .448726
**P VALUE**	0.000	0.689	0.723	0.386	0.593	0.698	0.817
**Model 8.** **Dependent variable:** thickness of AT T2, lower lateral third of the leg							
**Coefficient**	.912219	-.082109	-.6271101	-2.145115	-.0464108	.0168647	.2962078
**[95% conf. interval]**	.6575242 1.166914	-.5919729 .4277549	-3.34677 2.09255	-6.3001 2.00987	-.2383453 .1455237	-.0921114 .1258409	-.0556953 .6481108
**P VALUE**	0.000	0.741	0.637	0.295	0.620	0.751	0.095
